# Development of AMBER Parameters for Molecular Simulations of Selected Boron-Based Covalent Ligands

**DOI:** 10.3390/molecules28062866

**Published:** 2023-03-22

**Authors:** Maria Assunta Chiacchio, Laura Legnani, Enrico Mario Alessandro Fassi, Gabriella Roda, Giovanni Grazioso

**Affiliations:** 1 Department of Drug and Health Sciences, University of Catania, Viale A. Doria 6, 95125 Catania, Italy; 2 Department of Biotechnology and Biosciences, University of Milano-Bicocca, Piazza della Scienza 2, 20126 Milan, Italy; 3 Department of Pharmaceutical Sciences, University of Milan, Via L. Mangiagalli 25, 20133 Milan, Italy

**Keywords:** boron, covalent ligand, MD simulations, amber, force field, paramfit, β-lactamases

## Abstract

Boron containing compounds (BCCs) aroused increasing interest in the scientific community due to their wide application as drugs in various fields. In order to design new compounds hopefully endowed with pharmacological activity and also investigate their conformational behavior, the support of computational studies is crucial. Nevertheless, the suitable molecular mechanics parameterization and the force fields needed to perform these simulations are not completely available for this class of molecules. In this paper, Amber force field parameters for phenyl-, benzyl-, benzylamino-, and methylamino-boronates, a group of boron-containing compounds involved in different branches of the medicinal chemistry, were created. The robustness of the obtained data was confirmed through molecular dynamics simulations on ligand/β-lactamases covalent complexes. The ligand torsional angles, populated over the trajectory frames, were confirmed by values found in the ligand geometries, located through optimizations at the DFT/B3LYP/6-31g(d) level, using water as a solvent. In summary, this study successfully provided a library of parameters, opening the possibility to perform molecular dynamics simulations of this class of boron-containing compounds.

## 1. Introduction

Boron is an element widely distributed in nature. It plays essential functions, being involved in the growth, development, and metabolism of plants. Moreover, it is able to regulate vitamin D levels, help brain function, reduce the risk of developing cancer, and promote bone health in mammals [1]. Concerning this last aspect, it was demonstrated that diets deficient in boron hinder bone formation with respect to control diets that were supplemented with boron. Nevertheless, the exact mechanism behind this effect remains unclear [2].

The first isolated natural product, containing trace amounts of boron, was Boromycin. This is a macrolide that was isolated in the African soil from a strain of *Streptomyces antibioticus*. This antibiotic has been extensively studied for its therapeutic properties. In fact, it showed nanomolar potency against several HIV-infected cell lines [3] and against various bacterial strains, including *Mycobacterium tuberculosis* [4]. A compound, structurally related to Boromycin, is Aplasmomycin. It was isolated from *Streptomyces griseus* and has anti-plasmodium activity. In both the abovementioned natural products, the boron atom plays a structural role, causing the polyols to fold into compact structures. All these considerations suggest the increasing role that boron-containing compounds (BCCs) have assumed in scientific research.

Indeed, in the last years, BCCs have attracted growing attention because their clinical applications span from the treatment of fungal infection (i.e., tavaborole, Kerydin^®^, Pfizer, New York, NY, USA) [5] to the treatment of cancer (i.e., velcade, Bortezomib^®^, Janssen-Cilag International N.V., Olen, Belgium) [6]. Moreover, as evidence of their significant pharmacological activity, they also act as β-lactamase inhibitors (i.e., vaborbactam, in association with meropenem, Vaborem^®^, Menarini International Operation Luxembourg S.A., Luxembourg) [7], preventing the antibiotic-cleavage activity of antibiotic-resistant bacterial strains [8,9,10,11]. Additionally, they are able to form covalent complexes with other serine-proteases, such as chymotrypsin, trypsin, and thrombin [12]. Recently, some researchers have reported on the activity of BCCs in the inhibition of SARS-CoV-2 M^pro^, paving the way for the development of new therapeutic options to fight viral infections [13].

The warhead of BCCs can also react with organic compounds containing the hydroxide group, giving rise to a wide range of molecules endowed with biological activity [14]. In particular, they can irreversibly bound to structures containing *cis*-hydroxyl groups, such as riboflavin, pyridoxine, adenosine monophosphate, pyrimidine nucleotides, ascorbic acid, ribose, and polysaccharides [15].

One of the primary factors that drives the increasing use of BCCs in research and development was also their ability to switch between neutral trigonal planar sp2 and tetrahedral sp3 hybridization states. This property enables them to adopt various binding modes during the target recognition process and makes them attractive as high-affinity ligands with low molecular mass. Between BCCs, boronic acids can form different types of covalent adducts with nucleophiles in target proteins, including trigonal covalent, tetragonal covalent, or bidentate covalent adducts. These products are reversible, so unplanned covalent modifications of non-target proteins are minimized. The two hydroxyl groups, present in the chemical structure of boronic acids, offer six opportunities to form hydrogen bond contacts with amino acid residues, increasing the ligand’s affinity with the target protein. The ability of boron to alter its hybridization states enables boronate-based inhibitors to imitate the sp^2^ state of β-lactamase substrates, allowing them to efficiently bind to them. They can subsequently react with the nucleophilic serine of serine-β-lactamases or metal-β-lactamases to form the sp^3^ state, mimicking a high-energy intermediate. In the last case, the boron moiety can interact with metal ions in enzymes, which is useful in designing candidate drugs that can withstand selection pressures for drug resistance.

Despite the widely demonstrated pharmacological importance of BCCs, the appropriate molecular mechanics parameterization and the force fields, which allow us to perform computational studies essential for designing new ones and evaluate their conformational behavior, are not completely available to the researchers. Therefore, molecular dynamics (MD) simulations of BCCs, covalently bound to targets, can be performed only using computationally demanding QM-MM methods [16] or, as an alternative, by using the OPLS4 force fields (property of Schrödinger, LLC, New York, NY, USA) [17] available after the release of the commercial license.

In recent years, the parametrization of a limited number of aryl-, alkyl-boronic acids [12,18], and boronate esters [19] appeared in the literature. Tafi et al. [12] reported that the bonded, non-bonded, and point charges MacroModel/Amber force field was retrieved, in addition to GB/SA solvation parameters, for modeling boronic acids as tetrahedral adducts that are formed after coordination of the protease’s serine Oγ. The new force field was validated through flexible docking studies conducted on three crystallographic complexes of β-lactamases with boronic acids. The output of these studies matched up with the crystallographic conformation of the complexes as the global minimum energy structure. In another paper, Kurt et al. [18] generated the Amber force field parameters for benzodioxaboroles, a group of boron compounds with aromatic structures. Their study produced the necessary parameter library for performing molecular dynamics simulations of this class of compounds. In fact, the root mean square deviation (RMSD) value between the minimized geometries and the x-ray structures was found to closely match the values obtained by quantum mechanical calculations. Using an anti-cancer BCC as a ligand, the molecular dynamics (MD) simulation of the DNA-ligand complex was then successfully conducted, and the experimentally reported anti-cancer effect was confirmed by the simulations.

**Figure 1:**
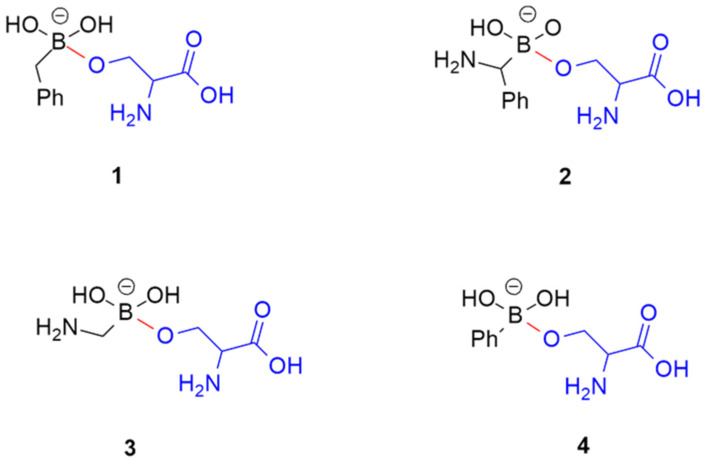
Chemical structures of the selected BCCs **1**–**4**. The atoms of the serine residue are colored in blue, whereas the covalent bond is depicted in red.

Despite these significant forward steps, to the best of our knowledge, the AMBER force field of phenyl-, benzyl-, benzylamino-, and methylamino-boronates are not available, even if these compounds are significant in numerous therapeutic fields. In fact, phenyl- and methyl-amino boronates are β-lactamases inhibitors [20] and anti-HIV agents [21]. Phenyl-boronates are also amazing prodrugs, like ZB483, able to produce a 40-fold increase in the endoxifen concentration peak in plasma when used in breast cancer therapy [22]. Based on their importance in medicinal chemistry, our attention has been focused on compounds **1**–**4**, reported in Figure 1, to potentially open the way to the search of new drugs with the fundamental support of molecular modeling.

With this aim and to cover the above reported computational gap, in this paper we calculated the molecular mechanics parameters of the representative BCCs **1**–**4**, using the Paramfit procedure, as suggested by AMBER developers [23]. The warheads of **1**–**4** were covalently bound to the side chain of a serine residue (in blue in Figure 1) to simulate the covalent bond (in red in Figure 1) to a putative serine-proteases. The accuracy of the retrieved parameters was established by performing MD simulations of the four BCCs in complex with AmpC β-lactamases, produced by *E. coli* [24]. Finally, to verify the new force field performance, the BCC torsional angles values, populated during the MD simulations, were compared with those found in the geometries located by a DFT conformational analysis [25].

## 2. Results and Discussion

The Paramfit procedure [23] was applied to compounds **1**–**4** to generate the dihedral parameters shown in Table 1, while the bond and angle parameters, reported in Table 2, were retrieved from the literature data [12,19]. In particular, the Paramfit procedure was applied to predict the parameters useful to simulate the covalent bond connecting the serine residue and the BCCs. In fact, in a standard AMBER simulation, in which a non-covalent ligand was simulated in complex with a serine-protease, the ligand is parameterized by GAFF [26] or the new version GAFF2 [27], while the biological counterpart is simulated by applying one of the force fields available in AMBER for simulating proteins, such as the ff14SB [28]. Conversely, when the bond between the ligand and the protein is covalent, the standard procedure, which uses the tleap module of AMBER to assign the force field parameters, fails. In fact, in this last case, the molecular mechanics parameters of the ligand-enzyme covalent bond (in red in Figure 1) are missing.

Moreover, improper dihedrals are also described in the same way [26,29], and the improper parameter, calculated through paramfit for our molecules, is reported in Table 1.

**Figure 2:**
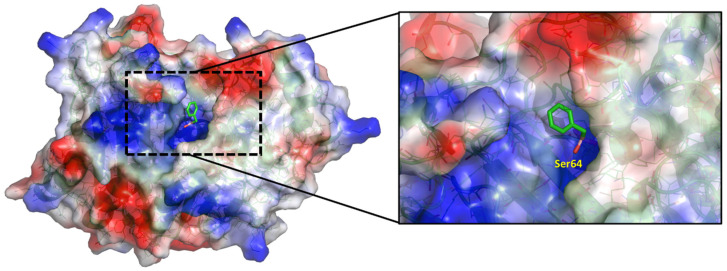
Binding mode of BCC **2** in the active site of AmpC, which resulted from docking calculations.

**MD simulations.** The above AMBER parameters, reported in Table 1 and Table 2, were used to accomplish the MD simulations of BCCs **1**–**4** in complex with the AmpC β-lactamase [24] in order to evaluate the conformational behavior of the single bonds around the Boron atom. Before starting MD simulations, BCCs **1**–**4**, deprived of the serine portion (colored in blue in Figure 1), were covalently docked by GOLD [30] into the active site of *E. coli* AmpC β-lactamase [24], adopting a computational procedure previously reported by us [9]. As an example, the binding mode of the BCC **2** is reported in Figure 2.

**Figure 3:**
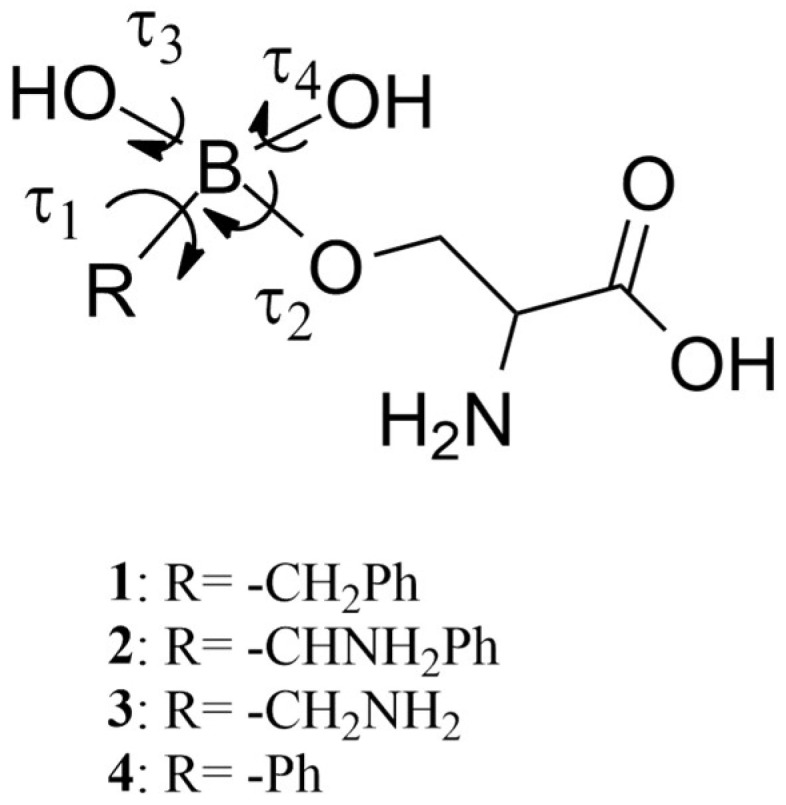
Torsional angles of compounds **1**–**4** evaluated during the MD simulations.

Then, MD simulations on the covalent complexes were performed, and the conformational freedom of the ligands was evaluated, observing the fluctuation of the dihedral angles τ_1_–τ_4_, (Figure 3 and Figures S1–S4, Supplementary Materials). Their average values during the simulations are summarized in Table 3.

**Figure 4:**
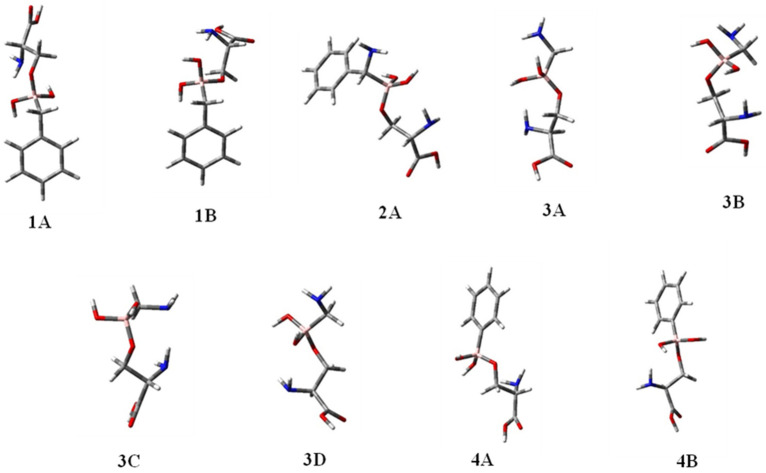
3D-plots of the significantly populated conformers of compounds **1**–**4** located by DFT calculations.

**Conformational analysis of BCCs 1–4.** With the aim of evaluating the accuracy of the new AMBER parameters developed by us, the full conformational analysis of **BCCs 1**–**4** (Figure 1) was performed through DFT calculations at the B3LYP/6-31G(d) level, using water as a solvent [31,32]. Successively, the torsional angles values τ_1_–τ_4_ in the located conformations of each compound (Table 4) were compared to those obtained by MD simulations (Table 3). The 3D plots of the most significant conformations of BCCs **1**–**4** are shown in Figure 4.

The attained results suggested that in the case of compound **1**, the minimum energy conformation **1A** shows geometrical preferences visited during the MD simulations. In fact, τ_1_, τ_2_, τ_3_, and τ_4_ have values of 171°, 142°, −68°, 176° in **1A**, fitting well with regions showing average values of 150°, 175°, −65°, 152°, for τ_1_, τ_2_, τ_3_, and τ_4_, respectively (see Table 4 and Figure 4).

For compound **2**, the preferred geometry **2A**, found during the DFT analysis, is the only significantly populated one (>90%). Its dihedral angle values (τ_1_ = −76°, τ_2_ = 155°, τ_3_ = −38°, τ_4_ = −102°) fitted well with the average values visited during the MD simulations (τ_1_ = −69°, τ_2_ = 176°, τ_3_ = −36°, τ_4_ = −49°), except for τ_4_, which is a bit underreported.

Once again, in the case of compound **3**, the preferences determined through the MD simulations correspond to the conformational ones found in the minimum energy conformer **3A** of the DFT analysis, which accounts for more than 50% of the population. In fact, τ_1_, τ_2_, τ_3_, and τ_4_ have values of −174°, 173°, 30°, −135°, respectively, in **3A**, corresponding to the regions of MD simulations described by average values of −155°, 176°, 49°, and −169° for the same dihedrals.

Finally, considering compound **4**, the minimum energy conformer **4A**, populated for 69.9% and with τ_1_, τ_2_, τ_3_, and τ_4_ of −139°, 177°, 50°, 95°, respectively, shows a very good correspondence with the MD data. In fact, during the MD simulations, dihedral angles τ_1_, τ_2_, and τ_3_ result and become −150°, 175°, 59°, respectively, while τ_4_ is 56° and 133°. The dihedral τ_4_ = 95°, found in the preferred conformation **4A**, could be considered the average of these two values.

**Figure 5:**
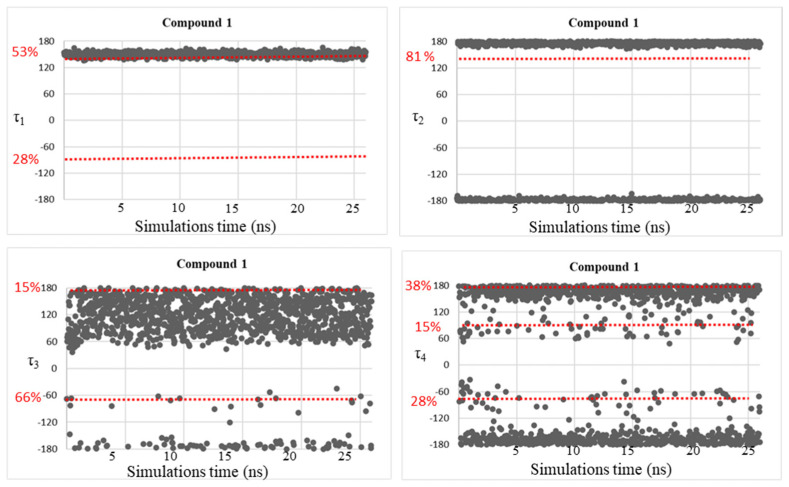
Dihedral torsion angle values τ_1_, τ_2_, τ_3_, and τ_4_ of compound **1** over the MD simulations. The red dotted line highlights the torsional angles values, found by DFT calculations, in the most populated energy minima. The red label on the left displays the corresponding population percentage.

**Figure 6:**
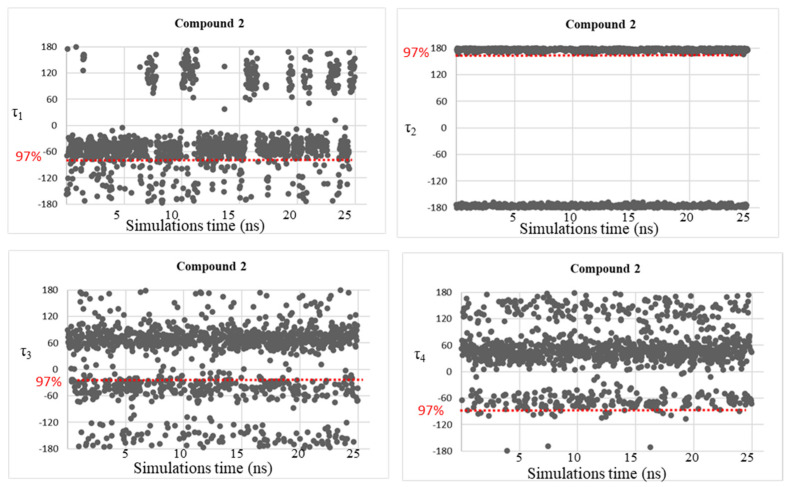
Dihedral torsion angle values τ_1_, τ_2_, τ_3_, and τ_4_ of compound **2** over the MD simulations. The red dotted line highlights the torsional angles values found by DFT calculations in the most populated energy minima. The red label on the left displays the corresponding population percentage.

**Figure 7:**
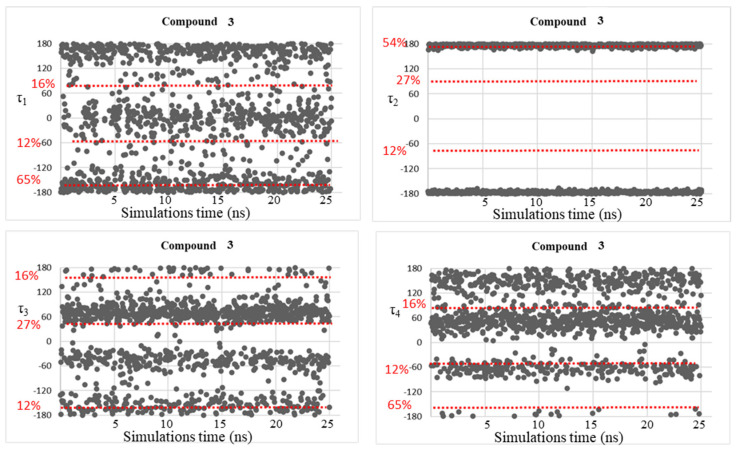
Dihedral torsion angle values τ_1_, τ_2_, τ_3_, and τ_4_ of compound **3** over the MD simulations. The red dotted line highlights the torsional angles values found by DFT calculations in the most populated energy minima. The red label on the left displays the corresponding population percentage.

**Figure 8:**
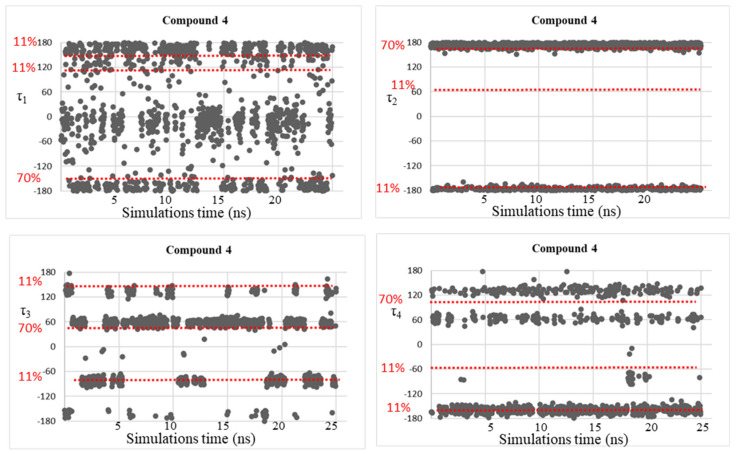
Dihedral torsion angle values τ_1_, τ_2_, τ_3_, and τ_4_ of compound **4** over the MD simulations. The red dotted line highlights the torsional angles values found by DFT calculations in the most populated energy minima. The red label on the left displays the corresponding population percentage.

Despite the good correspondence between the data attained by MD simulations and those of the DFT study, as explained above, all the free energy minima found by the conformational analysis studies were not fully visited during the MD simulations, considering the geometries populated by more than 10%. This fact might be due to the plausible steric clash between the ligand atoms and the residue atoms that can be found in the AmpC β-lactamases. Therefore, trying to solve this concern, MD simulations were accomplished on simplified systems constituted by the four investigated BCCs **1**–**4**, in which dihedral angle fluctuations over the MD simulations time (25 ns) were observed and reported in Figure 5, Figure 6, Figure 7 and Figure 8.

As expected, the conformational mobility of the boronic moieties significantly increased and new torsional angles were visited over the MD simulations, especially for dihedrals τ_3_ and τ_4_, describing the orientation of hydroxyl groups. Moreover, despite the absence of AmpC residues and the potential creation of steric hindrance or hydrogen bonds limiting the conformational freedom of the moiety, in the case of dihedral τ_2_, only the trans orientation resulted to be visited for compounds **3** and **4**.

These results represent a further confirmation of the good quality of the new determined parameters.

## 3. Materials and Methods

**Paramfit procedure.** Paramfit [23] is a program of the Amber package able to generate or improve force field parameters. Preliminarily, RESP atomic charges were calculated for all studied compounds, using the Gaussian16 program package [33] at the B3LYP level of calculation with a 6-31G(d) basis set [34]. Four different steps were followed to determine the requested parameters.

*Step 1.* Firstly, the system setup was formulated. A topology file, with the parameters to be fit, was created. Moreover, a frcmod file (or force field modification) was prepared containing all parameters to be fit.

*Step 2.* At this point, different compound conformations were generated. In particular, a variety of molecular structures that sampled the conformational space, involving parameters that had to be fit, were systematically generated. The quality of parameter set was controlled to verify that structures adequately sampled the space. The structure set quality was then evaluated. Quantum calculations were conducted to determine the energies of the different geometries. In this step, either the energy or forces of each conformer at the quantum level of theory B3LYP/6-31G(d) [34], already used above, were calculated, finding a list of ab initio quantum energies. The final energy values were extracted into a quantum energy data file, showing the energy of each structure in the same order as the coordinate file.

*Step 3.* Quantum output files were processed. Using paramfit and the energies file, parameters for force field equations were determined. When fitting to calculated energies, Paramfit derives parameters performing the least squares. In this way, the program is able to regulate the parameters. This procedure allows us to minimize the least squares difference between the quantum energy of the starting geometries and the determined AMBER energy. To this aim, the following equation is applied:

$$f\left(N,\;E_{QM},\;K\right)=\sum_{i=1}^{N}[{\left(E_{MM}\left(i\right)-E_{QM}\;\left(i\right)+K\right)}^{2}]$$

In this equation the different terms are: *N*, which is the number of geometries located for the molecule; *E_QM_*, which is the quantum energy evaluated through single-point calculations on the different conformations; and *E_MM_*, which is the calculated AMBER energy value for the same geometries. The constant term *K*, which depends on the molecule and set of input structures, was thus calculated. Contextually, R^2^ was determined.

*Step 4.* Finally, parameters to fit were defined and a new frcmod file was prepared, before starting the MD simulations.

**Docking and MD simulations.** Initially, the BCCs **1**–**4** were deprived of the structural moieties mimicking the serine residue. Covalent docking calculations were then performed by GOLD (version 2021.3, CCDC), using as a target the AmpC β-lactamases of *E. coli* (PDB accession code 1KE3) [24]. The ligand binding site was defined selecting residues included in a sphere with a radius of 12.0 Å from the side chain oxygen atom of Ser64 [5], and all residues were kept rigid in these calculations. The water molecules found in the X-ray were removed to avoid any steric clashes during this preliminary step of simulations. The pose acquiring the highest GoldScore was selected to generate the ligand-enzyme complexes for MD simulations. The RESP atom charges were assigned to the ligands by calculations at QM level using Gaussian16 [33] at the DFT/B3LYP/6-31G(d) level of theory [25,34]. The molecular mechanics parameters needed for MD simulations were assigned to the ligand-protein covalent complexes by the antechamber module of AMBER18 [29]: the ff14SB force fields [35] and TIP3P model [36] were used to represent the enzyme and the water solvent, respectively. In this step, a solvent box with a minimum distance of 15 Å from the AmpC surface was built for each complex. Prior to starting the MD simulations production runs, a minimization of the bulk solvent molecules was performed by applying a gradient criterion convergence of 0.2 kcal mol^−1^ Å^−1^. The entire systems were then optimized by a convergence criterion of 0.0001 kcal mol^−1^ Å^−1^. Successively, the whole systems were equilibrated, gradually increasing the temperature from 0 to 300 K over 60 ps of MD simulations in isocore conditions (NVT). Finally, production runs of 50 ns were accomplished for each ligand/enzyme complex in isothermal-isobaric ensemble at 300 K, with a 1 fs time-step (NPT). In these simulations, the systems were performed in periodic boundary conditions, while the Van der Waals and short-range electrostatic interactions were estimated within an 8 Å cutoff. The attained trajectory frames were visually inspected by VMD software [37]. MD simulations on compounds **1**–**4** (without the protein environment) were accomplished adopting the protocol here reported for the ligand/AmpC complexes.

**DFT Calculations.** All the calculations were carried out by using the GAUSSIAN16 program package [33]. The conformational space of compounds **1**–**4** was explored through the optimization of all the possible starting geometries using the DFT approach at the B3LYP level with the 6-31G(d) basis set [38,39]. To take into account the influence of the solvent, optimizations were conducted in water, using the polarizable continuum model (PCM) [40]. All the degrees of conformational freedom were considered. The conformational preferences of the single bonds of the molecules were determined through the evaluation of the three different *gauche*, *mgauche* and *anti* orientations, combining them and locating different geometries. The percentage contribution of each optimized conformation to the overall population was determined at 298 K through the Boltzmann equation. Vibrational frequencies were computed at the same level of theory to verify that the optimized structures were *minima*.

## 4. Conclusions

In this paper, we have developed the Amber force field parameters for phenyl-, benzyl-, benzylamino-, and methylamino-boronates, a group of BCCs capable of creating covalent complexes with the reactive amino acids (like serine, threonine, or cysteine) located in the active sites of enzymes like β-lactamases. In fact, the computational design of new compounds of this class required an accurate molecular mechanics parametrization that is only available for a limited number of aryl-, alkyl-boronic acids, and boronate esters. By our calculations, we have developed the force field parameters for selected BCCs **1**–**4**, testing them by performing MD simulations on ligand/β-lactamases covalent complexes (obtained by docking studies) and also by simulating them covalently bound with a putative serine residue. The robustness of our results was highlighted by the comparison of the values populated by torsional angles over the trajectory frames with those determined through a complete DFT conformational analysis. The good correspondence achieved the goal to create a library of parameters to perform MD simulations on new, selected BCCs that have important clinical applications and attracted the interest of medicinal and organic chemists.

## Figures and Tables

**Table 1:** Generated dihedral Amber parameters for compounds **1**–**4**, using the Paramfit procedure. For other dihedrals of the molecules, General Amber Force Field (GAFF) wildcard parameters were used. The atom names are indicated as in the GAFF/GAFF2 and ff14SB force field.

Dihedral	Divider	Vn (kcal/mol)	γ	n
oh-b-ca-ca	1	15.7206	170.366	−1.001
b-c2-ca-ca	1	0.7000	180.000	2.000
b-o-c3-c3	1	3.4800	106.880	1.500
b-o-c3-h1	1	4.1053	256.511	2.000
o-b-o-ho	1	0.8361	0.000	3.000
o-b-c2-ha	1	2.5070	172.978	0.129
o-b-c2-ca	1	15.7206	170.366	−1.000
o-b-o-c3	1	0.8347	0.000	3.000
ho-o-b-c2	1	2.0509	112.044	5.000
c2-b-o-c3	1	2.3740	114.599	−0.197
cx-2c-oh-b	1	3.4800	106.881	1.500
ho-oh-b-o	1	0.8361	0.000	3.000
ho-oh-b-c2	1	2.0509	112.044	5.000
ho-oh-b-ca	1	2.0509	112.044	5.000
oh-b-o-ho	1	0.8361	0.000	3.000
oh-b-oh-ho	1	0.8361	0.000	3.000
oh-b-c2-ha	1	2.5070	172.978	0.129
oh-b-c3-h1	1	2.5070	172.978	0.129
oh-b-c2-ca	1	15.7206	170.366	−1.000
oh-b-c3-ca	1	15.7205	170.366	0.000
h1-2c-oh-b	1	4.1053	256.511	2.000
2c-oh-b-o	1	2.3743	114.599	−0.197
2c-oh-b-c2	1	2.3743	114.599	−0.197
h1-2c-oh-b	1	4.1053	256.511	2.000
oh-b-c3-n3	1	−2.2276	0.000	2.156
2c-oh-b-oh	1	−44.4201	0.000	1.251
2c-oh-b-c3	1	−12.2011	0.000	2.000
ho-oh-b-c3	1	8.0608	0.000	−0.101
oh-b-c3-h1	1	0.5691	0.000	2.385
oh-b-ca-ca	1	15.7206	170.366	−1.001
2c-oh-b-ca	1	2.3743	114.599	−0.197
b-ha-c2-ha	Improper	1.1000	180.000	2.000

**Table 2:** Bond and angle Amber force-field parameters taken from literature data for compounds **1**–**4**.

Bond	K_r_ (kcal (mol·Å^2^) ^−1^	r_eq_ (Å)
b-o *	450.00	1.510
oh-b *	450.00	1.510
b-c2	340.00	1.630
b-ca	340.00	1.630
b-c3	326.80	1.510
**Angle**	**K_θ_** **(kcal/(mol·radian^2^)**	**θ_eq_** **(°)**
ca-c2-ha	47.90	123.30
b-o-ho *	35.00	109.50
ho-oh-b *	35.00	109.50
b-c2-ha **	50.00	109.50
b-c3-h1 **	50.00	109.50
b-c3-hc **	50.00	109.50
b-c3-c3 **	50.00	109.50
b-c3-n3 **	50.00	109.31
b-c2-ca **	127.38	120.97
b-ca-ca **	127.38	120.97
b-o-c3 *	90.00	109.50
o-b-o *	90.00	109.50
oh-b-o *	90.00	109.50
oh-b-oh *	90.00	109.50
oh-b-c2 *	60.00	109.50
oh-b-ca *	60.00	109.50
oh-b-c3 *	60.00	109.50
o-b-c2 *	60.00	109.50
2c-oh-b *	60.00	109.50
b-c3-ca **	127.38	111.90

* Data derived from ref. [8]; ** Data derived from ref. [15]

**Table 3:** Average values of dihedral angles τ_1_–τ_4_ for BCCs **1**–**4**, which resulted from MD simulations.

Compound	τ_1_ (°)	τ_2_ (°)	τ_3_ (°)	τ_4_ (°)
**1**	150	−177, 175	−169, −65, 54, 179	−164, 152
**2**	−154, −69, 88, 143	−176, 176	−36, 49	−49, 58, 154
**3**	−155, −28, 87	−176, 176	−138, −44, 49	−169, −58, 52, 151
**4**	−150, 153	−175, 175	−137, 59	−165, 56, 133

**Table 4:** Geometrical features, relative energies, and equilibrium percentages in water of the located conformations of compounds **1**–**4**.

	ΔE (kcal/mol)	%	τ_1_ (°)	τ_2_ (°)	τ_3_ (°)	τ_4_ (°)
**1A**	0.00	37.9	171	142	−68	176
**1B**	0.18	28.0	−71	154	−42	−85
**1C**	0.54	15.1	174	141	177	72
**1D**	0.91	8.1	−65	173	−142	−63
**1E**	1.25	4.6	−66	−70	−62	−178
**1F**	1.34	4.0	177	158	−43	−80
**1G**	2.28	0.8	68	−166	−146	−79
**1H**	2.32	0.8	171	−76	170	−51
**1I**	2.39	0.7	−64	−70	−65	−178
**2A**	0.00	96.5	−76	155	−38	−102
**2B**	2.16	2.5	−156	−146	−91	−147
**2C**	2.97	0.6	−69	−71	−54	−162
**2D**	3.54	0.2	75	−78	−136	−167
**2E**	4.10	0.1	−71	−73	−56	−162
**3A**	0.00	53.7	−174	173	30	−135
**3B**	0.73	15.6	64	38	170	111
**3C**	0.90	11.7	−59	−68	−168	−48
**3D**	0.91	11.6	−169	74	26	−161
**3E**	1.33	5.7	75	−144	−97	−151
**3F**	2.18	1.4	174	−170	−72	−142
**3G**	2.93	0.4	173	−77	−149	−160
**4A**	0.00	69.9	−139	177	50	95
**4B**	1.12	10.6	159	−171	−74	−143
**4C**	1.12	10.5	37	68	170	−57
**4D**	1.21	9.0	103	−70	−153	178

## Data Availability

Supporting data are available in the Supplementary Materials.

## Supplementary Materials

The following supporting information can be downloaded at: https://www.mdpi.com/article/10.3390/molecules28062866/s1, Figures S1–S4: History of τ_1_, τ_2_, τ_3_, and τ_4_ in **1**–**4** over the MD simulations starting from the docking pose.
