# Supplementary material for: Development of AMBER Parameters for Molecular Simulations of Selected Boron-Based Covalent Ligands
Source: Molecules. 2023 Mar 22;28(6):2866. doi: 10.3390/molecules28062866 (PMC10057150; doi:10.3390/molecules28062866)
Supplement: Supplementary file 1 [file molecules-28-02866-s001.zip › molecules-2288462-supplementary.pdf]

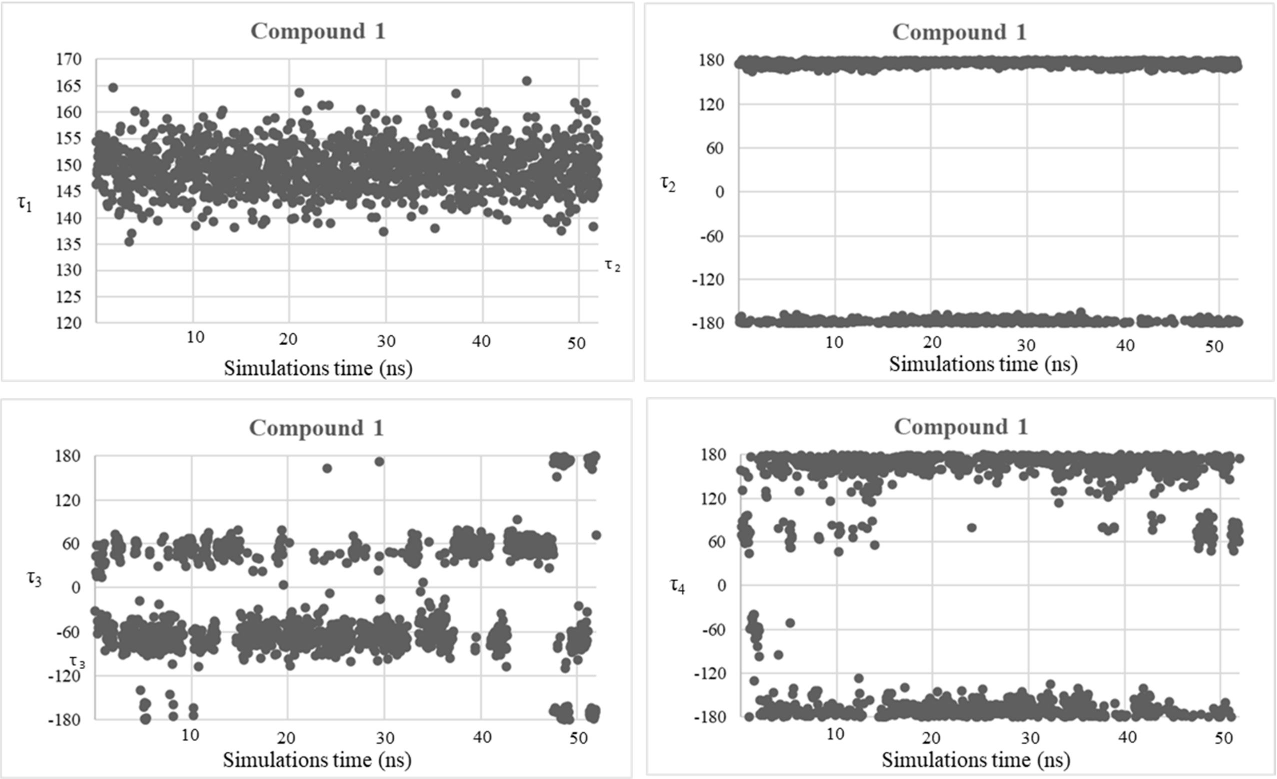


**Figure S1**. History of τ1, τ2, τ3, and τ4 in **1** over the MD simulations starting from the docking pose.

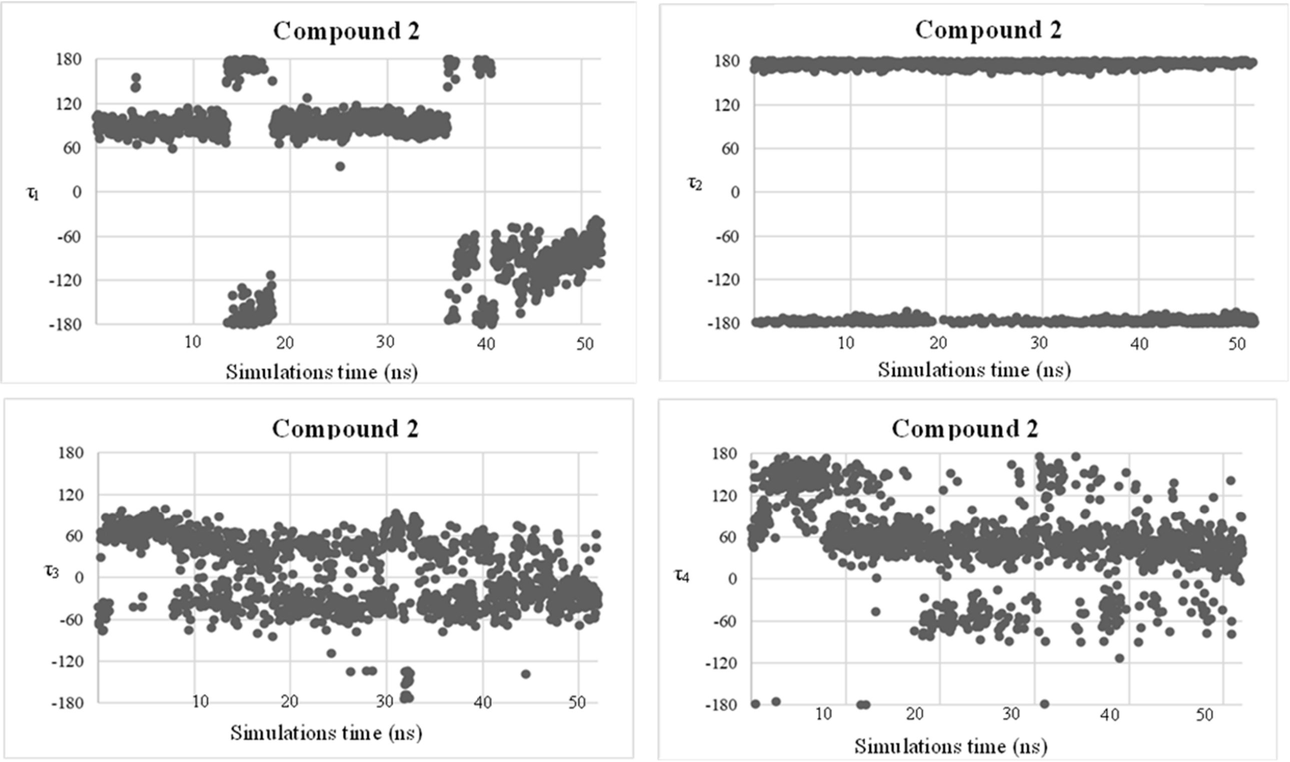


**Figure S2**. History of τ1, τ2, τ3, and τ4 in **2** in the MD simulation starting from the docking pose.

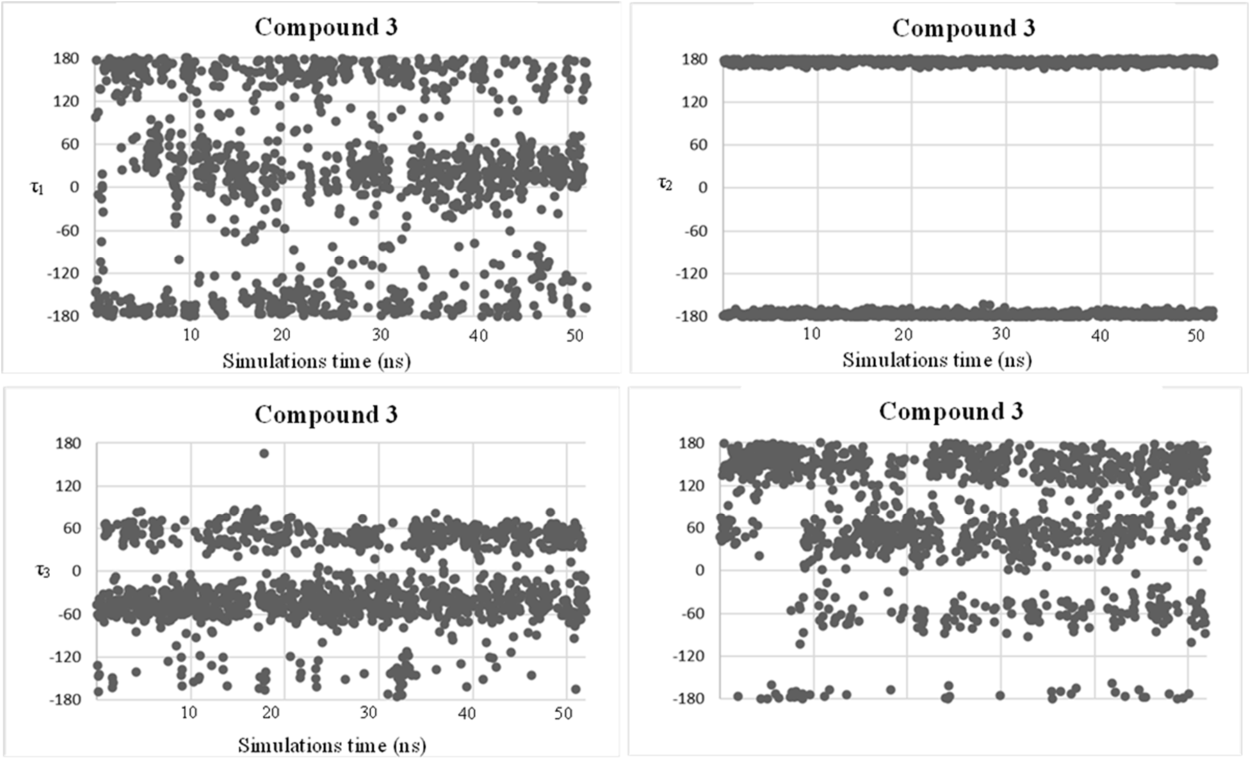


**Figure S3**. History of τ1, τ2, τ3, and τ4 in **3** over the MD simulations starting from the docking pose.

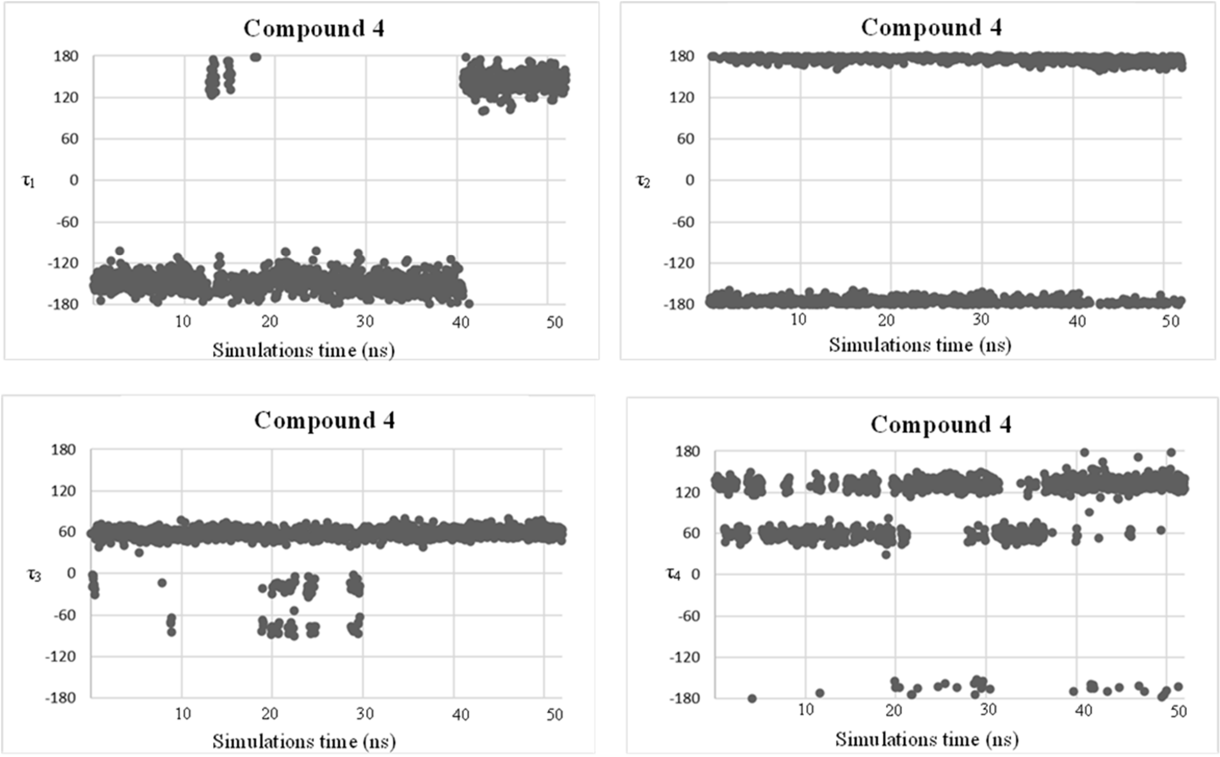


**Figure S4**. History of τ1, τ2, τ3, and τ4 in **4** over the MD simulations starting from the docking pose.
